# The news of treatment of variceal upper gastrointestinal bleeding

**Published:** 2011-11-24

**Authors:** N Tiuca, W Sztogrin

**Affiliations:** University Emergency Hospital, BucharestRomania

**Keywords:** stabilization of the patient’s hemodynamic status, severe liver disease, encephalopathy, rebleeding, mortality

## Abstract

Variceal bleeding is one of the dreaded complications of portal hypertension. Although its prognosis has improved over the last several decades, it still carries substantial mortality. Although most portal hypertensive bleeds result from the ruptured distal esophageal varices, bleeding from other sources such gastric varices, portal hypertensive gastropathy, and ectopic varices can lead to clinically significant bleeding.

Variceal bleeding typically presents as massive gastrointestinal (GI) bleeding with hematemesis, melena or hematochezia. In general, the terapeutic aims of management are to initially correct hypovolemia, to control bleeding, to prevent complications of bleeding, such as infection and renal failure and to prevent early rebleeding.

The treatment of bleeding esophageal varices differs substantially foom the treatment of other lesions of the upper gastrointestinal tract. Moreover, patients with esophageal varices typically have severe liver disease and thus are likely from poor nutrition, blood clotting disorders, and encephalopathy, all of which can adversaly affect morbidity and mortality.

## Introduction

When patients who have cirrhosis present with GI bleeding, they should be resuscitated and receive vasoactive agents such as: octreotide or vasopressin. All patients with variceal upper gastrointestinal bleeding should undergo upper endoscopy, performed after the patient is hemodynamically stable. Endoscopy should be performed promptly to diagnose the source of bleeding and to provide endoscopic therapy (preferably banding). The currently available treatment for acute variceal bleeding provides hemostasis in most patients.

### Stabilization

The hemodynamic instability related to hypovolemia is a common presentation of acute variceal bleeding. Therefore, patients require prompt resuscitation, hemodynamic support, and correction of hemodynamic dysfunction, which usually requires intensive care unit monitoring [**1**]. Judicios transfusion of blood products is necessary. Hemoglobin between 9 and 12g/dl is the target when transfusing cirrhotic patients who have variceal bleeding. In actively bleeding patients, platelets are transfuzed if the platelet count is under 50.000/ul. Fresh frozen plasma is administered for actively bleeding patients with a coagulopathy and an INR>1,5.

**Endotracheal intubation** for airway protection should be considered in anyone who is at risk for aspiration or is uncooperative.

**Renal failure** is a common complication of cirrhotic patients hospitalized for variceal bleeding [**2**]. The cause is typically multifactorial, including prolonged hypovolemia, overuse of diuretics, infection and hepatorenal syndrome. Renal failure is an independent risk factor for in–hospital mortality. Therefore, every effort should be made to avoid the development of renal failure by early aggresive resuscitation of patients and by avoiding nephrotoxic agents such as aminoglycosides and nonsteroidal drugs.

Recent studies have shown the importance of using **prophylactic antibiotics** in cirrhotic patients with bleeding. Bacterial infections are more common in cirrhotic patients with variceal bleeding (35% to 66%) then in noncirrhotic hospitalized patients (5% to 7%) [**3**]. Two factors have been identified to increase the risk of bacterial infections: severity of the liver disease and GI hemorrhage. In patients who have cirrhosis with variceal hemorrhage, prophylaxis against a bacterial infection reduces variceal rebleeding and improves survival [**4**]. The most common causes of bacterial infections in patients who had cirrhosis, with variceal bleeding, included spontaneous bacterial peritonitis, urinary tract infection and pneumonia. Typically gram–negative organisms are isolated. Therefore, antibiotics (oral quinolones or intravenous cephalosporins) should be given for 7 days in patients who have cirrhosis with bleeding [**5**].

As a part of the initial stabilization, **balloon tamponade with a Sengtaken–Blakemore** tube may be necessary to control brisk bleeding. Ballon tamponade succesfully achieves hemostasis in 90% of the cases of bleeding varices, but it has a high recurrence rate for rebleeding once the balloon is deflated.

### Vasoactive agents

Vasoactive agents for treating bleeding esophageal varices first were described in 1962. **Vasopressin** was the first agent studied because of its ability to induce splanchnic vasoconstriction, which leads to a decrease in portal inflow and portal pressure. Vasopresin’s cardiovascular adverse effects, such as myocardial ischemia and infarction, were limited in use.

**Somatostatin** is a natural peptide that induces splanchnic vasoconstristion which leads to a decreae in portal pressure. In four unblinded randomized studies, compared with placebo, somatostatin showed a trend toward benefit, with an overall risk reduction by 17% [**6**]. When compared with vasopressin, somatostatin was equivalent in terms of efficacy in controlling bleeding but had significantly fewer adverse effects [**7**]. A study by Villaneuva [**8,9**] compared somatostatin alone with combinated therapy of somatostatin and scerotherapy. This study observed the greatest benefit of combinated therapy somatostatin and scperotherapy. Unfortunately, somatostatin is not available in the United States.

**Octreotide**, a somatostatin analog is available in United States. It has similar properties as somatostatin, but with a longer biological half–life. Results regarding its efficacy compared with placebo, sclerotherapy, and balloon tamponade have been inconsistent. Many of the studies were small, low quality, and unblinded. A recent meta–analysis [**10**] demonstrated that octreotide was superior to other alternative therapies (placebo, vasopressin/terlipressin or sclerotherapy) in controlling acute variceal bleeding. Because of its excellent safety profine, octreotide has an added benefit that it can be administered in outside of the intensive care unit setting.[**11**] Continuous intravenous infusion of octreotide (100ug bolus, followed by 50–100ug/h) reduces splanchnic blood flow and portal blood pressures and is effective in the initial control of bleeding. It is administered promptly to all patients with active upper gastrointestinal bleeding and evidence of liver disease or portal hypertension until the source of bleeding can be determined by endoscopy.

**Terlipressin** is a long acting triglycyl–lysine derivative of vasopressin. It is transformed slowly to vasopressin by enzymatic cleavage. Because of this slow release to the active agent, terlipressin has significantly fewer adverse effects then vasopressin. Terlipressin is the pharmacologic therapy that has been shown to reduce mortality in acute variceal hemorrhage compared with placebo [**12**]. Unfortunately, terlipressin is not available in the United States.

### Endoscopic management

Both **sclerotherapy and band ligation** are very effective in controlling acute esophageal variceal bleeding and preventing rebleeding during the hospitalization [**13,14**]. These two modalies are the mainstay of therapy, and they are successful in achieving hemostasis in 80% to 90% of the patients with acute variceal bleeding [**15**]. The advantages of sclerotherapy include its ease of use, especially during massive bleeding, and its lower cost. Sclerotherapy, however, has been associated with ulceration and bleeding, bacteremia and stricture formation [**16,17**]. Band ligation has a lower rate of complication, but it can be difficult to use during acute bleeding [**19**]. A more recent study by Avgerinos and colleagues demonstrated what after initial control of bleeding; band ligation had significantly fewer rebleeding rates and complications, and it a achieved eradication with fewer endoscopic sessions than sclerotherapy [**20**]. Therefore, wherever feasible, band ligation should be the first-line endoscopic therapy for acute variceal bleeding [**21,22**]

### Transjugular Intrahepatic Portosystemic Shunt (TIPS)

TIPS is indicated in situations when acutely bleeding varices are refractory to medical therapy. TIPS has been shown in this situation to control bleeding in 95% of cases with a rebleed rate of only 18%. Furthermore, a study by Vangeli and colleagues reviewed 15 studies involving the use of TIPS to control bleeding when medical therapy failed. Similar to previous reports, bleeding was controlled in 93.6% of patients, and rebleed within 7 days was low (12.4 %) [**23**]. The mortality rate, however is between 30% and 40%. This is likely because patients with continued bleeding tend to be quite ill and have a high mortality rate despite any intervention.

### Surgery

Surgical options include selective portosystemic shunting, calibrated H grafts and devascularization procedures. The 30 – day mortality rate, however, approaches 80% with these procedures. Therefore, in most situations, surgical intervention for acutevariceal bleeding should be reserved for when medical thepay fails and TIPS is not available.

**Fig. 1:**
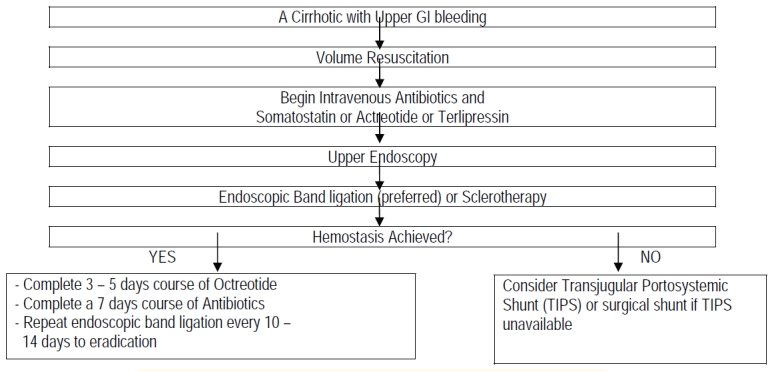
Suggested algorithm for managing acute variceal bleeding

**Fig. 1** outlines a reasonable algorithm for the management of acute variceal hemorrhage.

## Conclusions

Portal hypertension causes bleeding from varices (most commonly esophageal) or portal hypertensive gastropathy. Less than one–third of patients with portal hypertension and varices will develop acute bleeding. However, these lesions account for 10–20% of significant gastrointestinal hemorrhages, with a hospital mortality rate of 15–40%. If untreated, 50% will rebleed during hospitalization. When patients who have cirrhosis present with GI bleeding, they shoul be resuscitated and receive octreotide or other vasoactive agents. Endoscopy shoul be performed promptly to diagnose the source of bleeding and to provide endoscopic therapy (preferably banding). The currently available treatment for acute variceal bleeding provides hemostasis in most patients (90%).
